# Multiple PDE5Is use as a marker of decreased overall men’s health: A real-life study

**DOI:** 10.1371/journal.pone.0201601

**Published:** 2018-08-10

**Authors:** Davide Oreggia, Eugenio Ventimiglia, Paolo Capogrosso, Luca Boeri, Walter Cazzaniga, Filippo Pederzoli, Francesco Chierigo, Federico Dehò, Francesco Montorsi, Andrea Salonia

**Affiliations:** 1 University Vita-Salute San Raffaele, Milan, Italy; 2 Division of Experimental Oncology/Unit of Urology, URI, IRCCS Ospedale San Raffaele, Milan, Italy; 3 Department of Urology, University of Milan, Milan, Italy; Northwestern University, UNITED STATES

## Abstract

Erectile dysfunction (ED) is considered a sentinel marker for poor general men’s health status. Severe ED has been associated with poor response to phosphodiesterase type 5 inhibitors (PDE5Is) therapy. We sought to assess the association of multiple PDE5Is prescription with the overall patients’ health status. Socio-demographic and clinical variables from 939 consecutive white–European, heterosexual, sexually-active men seeking medical help for ED at same tertiary-referral academic outpatient clinic were analyzed. Health-significant comorbidities were scored with the Charlson Comorbidity Index (CCI). Patients have been stratified into naïve and non-naïve according to their history of previous prescriptions of any PDE5I. Every patient completed the International Index of Erectile Function (IIEF) questionnaire. Logistic regression models tested the association between patients’ baseline characteristics (thus including previous PDE5Is prescriptions) and the overall health status. Overall, 328 (35%) patients were non-naïve for PDE5Is. Of them, 172 (52%), 99 (30%), and 57 (17%) had been prescribed with 1, 2 or 3 different PDE5Is, respectively. Naïve and non-naïve patients did not differ in terms of age, BMI, baseline ED severity; conversely, non-naïve patients had a higher CCI score. At logistic MVA, the number of PDE5Is prescriptions emerged as an independent predictor of a higher burden of comorbidities regardless of ED severity; the higher the number of PDE5Is prescriptions, the higher the CCI score (OR 1.69, 2.49, and 2.90 for 1, 2 or 3 previous PDE5Is, respectively), after accounting for age, BMI, baseline ED severity and cigarette smoking. More than a third of patients seeking medical help for ED at a single tertiary-referral center were non-naïve for PDE5Is. The increasing number of previous prescriptions of PDE5Is emerged as a worrisome marker of a poorer overall men’s health status regardless of ED severity.

## Introduction

Most studies report an increase prevalence of erectile dysfunction (ED) as men age, reaching 20–40% in men between 60 and 69 years and up to 100% during 70s and 80s [1], depending on different definitions of ED itself among studies. In this context, many authors focused on the association between ED and other clinical conditions such as cardiovascular disease (CVD) [2–4], diabetes mellitus (DM), hypertension, dyslipidemia, obesity, metabolic syndrome (MetS), depression, chronic obstructive pulmonary disease (COPD) and lower urinary tract symptoms (LUTS) [3–5]. As a whole, ED emerged as an important sentinel marker of general health status [6,7], in particular considering CVD [8]. As a matter of fact, the evidence that ED usually precedes CVD, should offer the clinician a window period of opportunity for diagnosis and risk mitigation in the everyday clinical practice [9]. A similar picture has been observed for DM; indeed, data suggested that DM is a major risk factor for ED, as well as ED can be the presenting symptom of DM [10]. Besides all this, ED is also significantly associated with other comorbid conditions; for instance, data from the Massachusetts Male Aging Study pointed out that ED was predictive of the subsequent development of MetS [11]. Likewise, using Child-Pugh classification and serum albumin level another study identified a correlation between ED and liver impairment in patients with cirrhosis and chronic hepatitis [12]. Similarly, different studies showed a higher prevalence of COPD in patients with ED, and vice versa, if compared with general population [13].

Salonia et al. [6] stressed the value of ED as marker for lower general health status showing that the severity of ED, as objectively interpreted using the erectile function (EF) domain of the International Index of Erectile Function (IIEF), accounted for a higher Charlson Comorbidity Index (CCI), which objectively scores most of the potential comorbid conditions with a significant impact upon the overall prognosis of an individual, even regardless the cardiovascular burden [7]. Furthermore, others found that patients with ED have a higher risk of all-cause death as compared with the general population [14].

According to these findings, patients with severe ED may certainly deserve a comprehensive assessment of their general health status in order to identify potential unrecognized life-threatening conditions; however, a measure of ED severity, as assessed with a self-reported validated questionnaire (e.g. IIEF), could be unavailable at the time of the first office evaluation in the real-life clinical setting. In this context, as a first line therapy [15] phophodiesterase type 5 inhibitors (PDE5Is) are generally prescribed to the majority of patients with ED, with some of them usually trying at least two if not all currently available molecules [15]. This seems to happen because patients always virtually search for even more effective pills, with fewer side effects, or closer to their actual expectations. Of note, non-responders to PDE5Is are likely to harbor a severe organic EF impairment at baseline [15], eventually associated with a worse overall health condition.

Given that the history of previously prescribed ED medication can be easily and comprehensively assessed at the time of the first clinical evaluation for ED, we cross-sectionally sought to determine if multiple PDE5Is prescriptions could be used as a proxy of overall men’s health status in a cohort of white–European men seeking medical help for ED in a tertiary-referral academic center.

## Materials and methods

### Patients

From January 2010 to September 2015, socio-demographic and clinical variables from 939 consecutive white–European, heterosexual, sexually-active men seeking medical help for ED as a primary compliant at their first office visit at same tertiary-referral academic outpatient clinic were considered for this exploratory analysis.

For the specific purpose of this study, ED was defined as the persistent inability to attain and maintain an erection sufficient to permit satisfactory sexual performance [16]; moreover, patients were enrolled if they failed at least 50% of four consecutive attempts at sexual intercourse.

Patients were comprehensively assessed with a detailed sexual history; according to their history of previous use/prescription of any PDE5I, patients have been stratified into naïve and non-naïve and the number of different previously prescribed compounds was accurately recorded. Moreover, to provide a frame of reference for objectively interpreting psychometric data on sexual functioning, patients were invited to complete the IIEF domains [17]. To interpret ED severity, we used the IIEF-EF domain classification as proposed by Cappelleri et al [18].

Health-significant comorbidities were scored with the Charlson Comorbidity Index (CCI) both as a continuous or a categorized variable (i.e., 0 vs. ≥1) [19]. We used the *International Classification of Diseases*, 9^th^ revision, Clinical Modification (ICD-9-CM). Measured body mass index (BMI), defined as weight in kilograms by height in square meters, was considered for each patient. Recreational habits, including cigarette smoking history, and the practice of regular physical activity (PA), as defined for at least 2.5 hours a week, according to the American College of Sports Medicine and the American Heart Association [20], were also detailed for every patient.

Patients were thus segregated according to their educational status into a low educational level group (LL), which included patients with an elementary or secondary school education, and a high educational level (HL), which considered men with either high school degree and/or university/postgraduate degree.

Data collection followed the principles outlined in the Declaration of Helsinki. All patients signed an informed consent agreeing to supply their own anonymous information for future studies. The study was approved by the Ethical Committee of San Raffaele Institute, Milan (protocol N. 2014-outpatient clinic)

### Statistical analyses

The aim of the study was to to investigate whether the number of previous PDE5Is prescriptions was associated with a lower overall health status, as depicted with the CCI score; we developed univariable and multivariable logistic regression models, including as covariates age at diagnosis, BMI, cigarette smoking, stable sexual relationship, PA, IIEF-EF score, and the previous number of prescribed PDE5Is. Thereof, logistic regression analyses tested the association between clinical variables (including previous PDE5i prescriptions) and the presence of comorbidities (CCI>0). The adjusted probability of reporting a CCI>0 according to the baseline IIEF-EF score and the number of previously prescribed PDE5is was presented graphically. All statistical analyses were performed using R version 3.3.0 (R Foundation for Statistical Computing).

## Results

Table 1 lists the characteristics and descriptive statistics of the entire cohort of individuals and according to a formal stratification into naïve vs. non-naïve for previous PDE5Is prescriptions. The majority of patients were naïve for PDE5Is (65%); conversely, 328 (35%) patients reported previous use of one PDE5I at least. Of these latter, 172 (52%), 99 (30%) and 57 (17%) had been prescribed with 1, 2 or ≥3 different PDE5Is, respectively. Tadalafil emerged as the most prescribed PDE5I (254 patients), followed by sildenafil (180 patients), vardenafil (107 patients), and avanafil (5 patients).

**Table 1:** Characteristics and descriptive statistics of patients according to PDE5Is prescription (non-naïve vs. naïve).

	Non-naïve	Naive	Overall
Patients [No. (%)]	328 (34.9)	611 (65.1)	939
Age (years)			
Median	52	47	49
IQ Range	42–62	35–58	38–59
Age [No. (%)]			
<40	69 (21)	208 (34)	277 (29)
41–49	71 (22)	135 (22)	206 (22)
50–59	95 (29)	134 (22)	229 (24)
60+	93 (28)	134 (22)	227 (24)
BMI (kg/m^2^)			
Median	25	25	25
IQ Range	23–28	23–27	23–27
BMI [No. (%)]			
18–24.9	146 (45)	319 (52)	465 (50)
25–29.9	147 (45)	236 (39)	383 (41)
30+	35 (11)	56 (9)	91 (10)
CCI [No. (%)]			
CCI 0	220 (67)	505 (83)	725 (77)
CCI 1	48 (15)	47 (8)	95 (10)
CCI ≥2	60 (18)	59 (10)	119 (13)
Educational level [No. (%)]			
Low	232 (77)	480 (79)	734 (78)
High	96 (23)	131 (21)	205 (22)
Sexual relationship [No. (%)]			
Stable	254 (77)	440 (72)	672 (72)
Non-stable	74 (23)	171 (28)	267 (28)
Cigarette smoking [No. (%)]			
No/Never	195 (59)	376 (62)	571 (61)
Former	41 (12)	79 (13)	120 (13)
Current	92 (28)	156 (26)	248 (26)
PA [No. (%)]			
Regular	177 (54)	340 (56)	517 (55)
Non-regular	151 (46)	271 (44)	442 (45)
IIEF-EF (points)			
Median	16	19	18
IQ Range	7–23	8–26	7–25
IIEF-EF [No. (%)]			
<10	128 (39)	185 (30)	313 (33)
11–16	39 (12)	88 (14)	127 (14)
17–21	51 (16)	75 (12)	126 (13)
22+	110 (34)	263 (43)	373 (40)
Previous PDE5Is [No. (%)]			
0	0 (0)	611 (100)	611 (65)
1	172 (52)	0 (0)	172 (18)
2	99 (30)	0 (0)	99 (11)
≥3	57 (17)	0 (0)	57 (6)
Pre SIL [No. (%)]			
No	148 (45)	611 (100)	759 (81)
Yes	180 (55)	0 (0)	180 (19)
Pre TAD [No. (%)]			
No	74 (23)	611 (100)	685 (73)
Yes	254 (77)	0 (0)	254 (27)
Pre VAR [No. (%)]			
No	221 (67)	611 (100)	832 (89)
Yes	107 (33)	0 (0)	107 (11)
Pre AVA [No. (%)]			
No	324 (99)	611 (100)	935 (99)
Yes	4 (1)	0 (0)	4 (1)

Keys: BMI = body mass index; CCI = Charlson Comorbidity Index; PA = Physical Activity; IIEF-EF = International Index of Erectile Function–Erectile Function domain; SIL = Sildenafil; TAD = Tadalafil; VAR = Vardenafil; AVA = Avanafil

Most patients (61%) were no smokers (62% vs. 59% among naïve and non-naïve, respectively), had a stable sexual relationship (72% naïve vs. 77% non-naïve, respectively), and a high education level (79% naïve vs. 77% non-naïve).

Overall, 725 (77%) patients did not report health-significant comorbidities; among them, 67% and 83% were non-naïve and naïve patients, respectively. In contrast, a greater proportion of non-naïve men had one health-significant comorbid condition at least as compared with naïve individuals (33% vs. 18%).

Of clinical relevance, the higher the number of previous PDE5Is prescriptions, the greater the risk of high CCI score, both at univariable logistic regression and after adjusting for the aforementioned confounders (Table 2). In particular, the adjusted ORs turned out to be 1.69 (1.09–2.58) for patients with only one previous PDE5I prescription, versus 2.49 (1.50–4.09) and 2.90 (1.55–5.37) for those who did receive either 2 or ≥3 PDE5Is, respectively.

**Table 2:** Logistic (OR [95%CI]) regression models predicting a CCI≥1 in the whole cohort of patients (No. = 939).

	Number (events)	UVA OR [95% CI]	MVA OR [95% CI]
**Age (years)**			
** <40**	803 (141)	1.0 *	1.0 *
** 41–49**	82 (39)	4.26 [2.66–6.82]	3.59 [2.17–5.93]
** 50–59**	20 (11)	9.82 [4.78–21.37]	7.37 [3.46–16.54]
** 60+**	34 (23)	5.74 [2.33–14.48]	4.86 [1.88–12.84]
**BMI (Kg/m2)**			
** 18–24.9**	383 (102)	1.00 *	1.00 *
** 25–29.9**	465 (85)	1.62 [1.17–2.25]	1.57 [0.89–2.72]
** 30+**	91 (27)	1.89 [1.12–3.11]	1.35 [0.94–1.94]
**Educational level**			
** High**	734 (166)	1.00 *	1.00 *
** Low**	205 (48)	1.05 [0.72–1.50]	0.88 [0.44–1.82]
**Sexual relationship**			
** Non-stable**	267 (66)	1.00 *	1.00 *
** Stable**	672 (148)	0.86 [0.62–1.20]	0.64 [0.39–1.09]
**Cigarette smoking**			
** No/Never**	571 (133)	1.00 *	1.00 *
** Former**	120 (34)	1.30 [0.83–2.01]	1.31 [0.79–2.13]
** Current**	248 (47)	0.77 [0.53–1.11]	0.96 [0.63–1.43]
**Physical activity**			
** Non-regular**	442 (93)	1.00 *	1.00 *
** Regular**	517 (921)	1.08 [0.80–1.47]	1.40 [0.85–2.36]
**IIEF-EF (points)**			
** <10**	313 (115)	1.00 *	1.00 *
** 11–16**	127 (20)	0.61 [0.38–0.96]	0.37 [0.20–0.64]
** 17–21**	373 (46)	0.24 [0.16–0.35]	0.64 [0.38–1.04]
** 22+**	126 (33)	0.32 [0.19–0.54]	0.33 [0.22–0.49]
**Previous PDE5Is (number)**			
** 0**	611 (106)	1.00 *	1.00 *
** 1**	172 (46)	1.74 [1.16–2.58]	1.69 [1.09–2.58]
** 2**	99 (37)	2.84 [1.79–4.48]	2.49 [1.50–4.09]
** ≥ 3**	57 (25)	3.72 [2.10–6.53]	2.90 [1.55–5.37]

Keys: BMI = body mass index; CCI = Charlson Comorbidity Index; IIEF-EF = International Index of Erectile Function–Erectile Function domain; PDE5Is = Phosphodiesterase type 5 inhibitors

**Fig 1:**
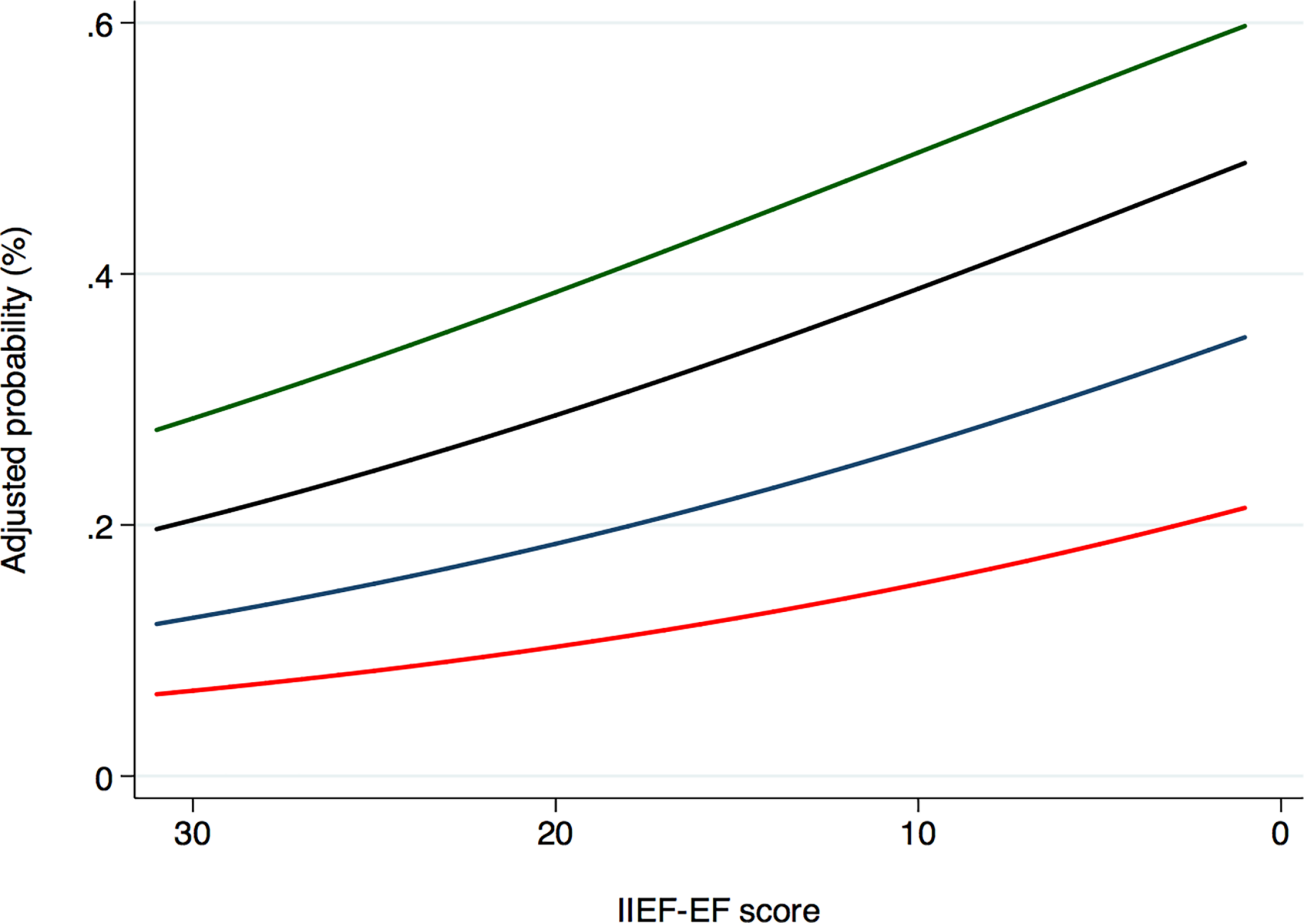
Adjusted probability of presenting comorbidities (CCI≥1) according to IIEF-EF baseline score. The green line represents patients with previously prescribed ≥3 PDE5Is; the black line represents patients with 2 previously prescribed PDE5Is; the blue line represents patients with 1 previously prescribed PDE5I; the red line represents patients näive for PDE5is.

Patients with the same baseline IIEF-EF score showed a higher adjusted probability of presenting with comorbidities (CCI≥1) (Fig 1); men with severe ED who received ≥3 PDE5Is had a probability of CCI≥1 of 54% (95%CI: 42–65) as compared with 43% (95%CI: 35–51), 30% (95%CI: 24–35) and 17%(95%CI: 13–22) for severe ED patients who previously received 2, 1 and 0 PDE5Is, respectively.

## Discussion

This study was designed to investigate a potential correlation between the number of previously prescribed PDEIs and the general health status in a relatively large and homogenous cohort of heterosexual, sexually active men seeking medical help for ED as their primary compliant. We observed that men with a higher number of previous prescriptions of PDE5Is had a greater CCI, thus supporting the clinical concept of a lower overall health status associated with the need of an increased number of different drugs. Of note, the risk that patients with ED also have relevant comorbidities appeared to be higher for those with an increasing number of previously prescribed PDE5Is, regardless of baseline ED severity. According to these findings, the history of previous oral ED medications may serve as a user-friendly tool to identify those patients deserving a comprehensive health assessment at the time of first office evaluation for ED.

Our interest was fuelled by growing evidence regarding the relationship between EF and overall men’s health status, and the even more discussed concept that ED per se could be a proxy of a worse male health as a whole [6,7,11,21]. To this regard, in a survey conducted on a cohort of 2,213 men with ED as compared with 11,065 matching controls, Chung et al. analysed the prevalence of 36 different comorbidities, showing an increased risk of metabolic disorders, gastrointestinal diseases and chronic pulmonary diseases for men with ED [21]. Similary, Salonia et al. [6] studied the correlation between ED severity and the general male health status in a cohort of 140 patients with new-onset ED in the real-life setting. Their findings showed a significant correlation between IIEF-EF scores and the burden of comorbid conditions as objectively interpreted with the CCI, pointing out that the degree of ED severity was an independent predictor for a worse health status, even after adjusting for other factors like patient’s age, BMI, cigarette smoking and PA. Overall, these findings outline the need to look at potential comorbid conditions among patients with more severe forms of ED; however, the application of validated instruments assessing ED severity, such as the IIEF questionnaire, is certainly not systematic among physicians, with data showing that in up to 44% of studies performed in high volume centers similar tools had not been even used to evaluate EF [22]; as a matter of fact, this would suggest that an even lower use has to be expected in the real-life clinical setting. Therefore we looked at the history of previously prescribed ED medications as a easily applicable, user-friendly parameter to interpret ED severity and identify those patients with a higher risk of presenting severe comorbidities.

To this aim, we subdivided the overall cohort of patients into naïve and non-naïve for PDE5Is and looked at the individual number of previous prescriptions of different compounds. Indeed, non-responders to PDE5Is are likely to shift to at least another same-class compound before being prescribed with a second line therapy [15]. At the same time, it is widely recognized that patients with a severe organic impairment of the erectile tissue are less likely to be satisfied after treatment with PDE5Is (any type) [23]. We reported that more than one out of three patients had received at least one previous prescription for PDE5Is at the time of first office assessment. This could be certainly due to the fact that the analyses were conducted in a tertiary-referral center and many patients come and seek a second opinion both because of a previous failure or since they are not completely satisfied with previous treatments. However, we consider this demographic aspect clinically important because it seems to suggest that our cohort of patients did actually represent the real-life scenario observed over the last five to ten years at least.

Our findings also showed that naïve and non-naïve men did differentiate neither for most of the well-recognized variables suggestive of ED severity (i.e., age and BMI) nor for their lifestyle and socio-demographic characteristics (thus including, cigarette smoking, PA and relationship status), although, non-naïve patients clearly depicted a worse overall health status as objectively scored with the CCI. To this regard, we should point out that our analyses were not designed to specifically study socio-demograhic differences associated with PDE5Is awareness.

Most relevant findings of these analyses were i) the baseline different ED severity between naïve and non-naïve patients—with the former being less severe than the latter, as it was clinically expected due to a potential lack of treatment effectiveness among those individuals who had decided to move from one PDE5I to another; and, ii) the greater CCI score in non-naïve men, thus indirectly pointing out the potential role of ED as a proxy of a lower overall men’s health. Therefore we provided novel evidence of the clinical relevance of an indirect user-friendly measure (namely, the number of previous PDE5Is prescriptions in a non-naïve individual seeking medical help for ED) to easily define overall men’s health status.

Our study is not devoid of limitations. First, this was a hospital‑based study, raising the possibility of a number of selection biases. Patients were recruited from a single tertiary-referral academic outpatient clinic; therefore, larger studies across different centers and populations will be needed to substantiate our findings. Second we used the datum of previously assumed PDE5Is as reported by patients without considering who prescribed them, treatment scheme, and the modality (correct or incorrect) of their eventual intake. Multiple PDE5Is prescriptions may depend on experienced side effects, on the availability of second/third line treatments and on care provider familiarity with them [14,15]: unfortunately, our analysis was not able to account for this possible confounder. However, the finding of a correlation between ED severity and the number of PDE5Is prescriptions would support the theory that at least in this cohort, a lower efficacy was the main drive to shift from one drug to the other. Finally as a cross-sectional study we are not able to test the risk of a further worsening of the health conditions associated with the investigated outcome; longitudinal study should be conducted to clarify this issue.

## Conclusions

This exploratory analysis showed that more than one third of patients seeking medical help for ED at a single tertiary-referral academic centre are non-naïve for PDE5Is. In this context, non-naïve patients depicted higher CCI scores, thus suggesting a lower overall health status. Moreover, the increasing number of previously prescribed PDE5Is emerged as a worrisome marker of increasingly poor men’s health status regardless of baseline ED severity. Thereof, these findings provide novel evidence of the potential clinical relevance of an indirect user-friendly measure (namely, the number of previous PDE5Is prescriptions) to easily identify patients deserving a more comprehensive medical assessment.

## Supporting information

S1 Appendix Master dataset. (CSV)
